# A Versatile Ionic Liquid Additive for Perovskite Solar Cells: Surface Modification, Hole Transport Layer Doping, and Green Solvent Processing

**DOI:** 10.1002/advs.202412959

**Published:** 2025-01-09

**Authors:** Seong‐Jin Jeong, Sung Hwan Park, Siwon Yun, Meng Qiang Li, Dasol Kim, Yongchan Kim, Yun Hee Chang, Jaewon Lee, Jongchul Lim, Tae‐Youl Yang

**Affiliations:** ^1^ Department of Materials Science and Engineering Chungnam National University 99 Daehak‐ro Yuseong‐gu Daejeon 34134 Republic of Korea; ^2^ Division of Advanced Materials Korea Research Institute of Chemical Technology (KRICT) 141 Gajeong‐ro, Yuseong‐gu Daejeon 34114 Republic of Korea; ^3^ Graduate School of Energy Science and Technology (GEST) Chungnam National University 99 Daehak‐ro, Yuseong‐gu Daejeon 34134 Republic of Korea; ^4^ Department of Chemical Engineering and Applied Chemistry Chungnam National University 99 Daehak‐ro, Yuseong‐gu Daejeon 34134 South Korea

**Keywords:** dopant, hole transport layer, ionic liquid, perovskite solar cells, surface passivation

## Abstract

Hole‐transport layers (HTL) in perovskite solar cells (PSCs) with an n‐i‐p structure are commonly doped by bis(trifluoromethane)sulfonimide (TFSI) salts to enhance hole conduction. While lithium bis(trifluoromethanesulfonyl)imide (LiTFSI) dopant is a widely used and effective dopant, it has significant limitations, including the need for additional solvents and additives, environmental sensitivity, unintended oxidation, and dopant migration, which can lead to lower stability of PSCs. A novel ionic liquid, 1‐(2‐methoxyethyl)‐1‐methylpyrrolidinium bis(trifluoromethylsulfonyl)amide (MMPyTFSI), is explored as an alternative dopant for 2,2′,7,7′‐tetrakis(N,N‐di‐p‐methoxyphenylamino)‐9,9′‐spirobifluorene (spiro‐OMeTAD). MMPy ions act as a surface passivator, reducing defects on the perovskite surface, while TFSI ions facilitate p‐type doping. MMPyTFSI functions as an efficient dopant, maintaining excellent performance even when tetrahydrofuran (THF) is utilized as a solvent in place of chlorobenzene (CB), while significantly reducing the environmental impact of the process. The optimized PSC achieves a power conversion efficiency (PCE) of 23.10% and demonstrates enhanced long‐term stability in all aging tests for over 1000 h in a humid atmosphere, at high temperature, and under simulated sunlight illumination. These results demonstrate that MMPyTFSI is an effective and environmentally friendly dopant for producing stable and efficient PSCs.

## Graphical abstract

The ionic liquid, 1‐(2‐methoxyethyl)‐1‐methylpyrrolidinium bis(trifluoromethylsulfonyl)amide (MMPyTFSI), is introduced as a dopant for 2,2′,7,7′‐tetrakis(N,N‐di‐p‐methoxyphenylamino)‐9,9′‐spirobifluorene (spiro‐OMeTAD) in perovskite solar cells. This dopant additionally induces surface passivation of the perovskite and could be used with the green solvent. The resulting power conversion efficiency is 23.10%, and long‐term stability under humid, high‐temperature, and light conditions for over 1000 h is achieved.

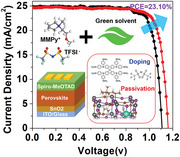

## 1 Introduction

The power conversion efficiency (PCE) of single‐junction perovskite solar cells (PSCs) has dramatically increased from an initial 3.8% to over 26%, highlighting their significant potential as a future renewable energy solution.^[^ ^1^, ^2^, ^3^, ^4^, ^5^ ^]^ The efficiency of PSCs is significantly influenced not only by the quality of the perovskite light‐absorbing layer, which is responsible for photon absorption and photogenerated charge carriers, but also by the characteristics and type of charge transport layers that are crucial for the efficient extraction of these carriers.^[^ ^6^, ^7^, ^8^, ^9^, ^10^, ^11^, ^12^ ^]^ Additionally, the charge transport efficiency and chemical stability of these layers are critical in determining the long‐term operational stability of PSCs.^[^ ^13^, ^14^, ^15^ ^]^

The highest efficiency of n‐i‐p perovskite solar cells (PSCs) has been reported with SnO_2_ as the electron transport layer (ETL) and 2,2′,7,7′‐tetrakis(*N,N*‐di‐p‐methoxyphenylamine)‐9,9′‐spirobifluorene (spiro‐OMeTAD) as the hole transport layer (HTL).^[^ ^16^ ^]^ After being introduced by H. Snaith and N.G. Park, spiro‐OMeTAD is the most widely used HTL material to fabricate high‐performance PSCs. Because spiro‐OMeTAD itself exhibits high series resistance and charge recombination within devices due to its low hole conductivity, lithium bis(trifluoromethane)sulfonimide (LiTFSI) is commonly added to improve the conductivity of spiro‐OMeTAD.^[^ ^17^, ^18^, ^19^, ^20^, ^21^ ^]^ The addition of bis(trifluoromethane)sulfonimide (TFSI) salts leads to the formation of spiro‐OMeTAD^+^TFSI^−^ radicals which generate hole carriers and improves band alignment between spiro‐OMeTAD and the perovskite, ultimately enhancing charge extraction ability.^[^ ^22^ ^]^

LiTFSI, while effective, suffers from several drawbacks that compromise the stability of PSCs. The high hygroscopicity of LiTFSI facilitates moisture ingress into the HTL, leading to subsequent permeation into the perovskite layer and accelerated degradation.^[^ ^23^, ^24^ ^]^ Additionally, the small size of lithium ions allows them to migrate easily into the perovskite layer, leading to their accumulation and resulting in decreased device performance and long‐term stability.^[^ ^18^ ^]^ The additional additive, 4‐*tert*‐butylpyridine (4‐*t*BP), to enhance LiTFSI solubility and ensure uniform spiro‐OMeTAD thin film formation introduces additional stability concerns under high‐temperature and high‐humidity conditions.^[^ ^25^, ^26^, ^27^, ^28^, ^29^ ^]^

To address these issues, introducing new dopants by replacing the Li^+^ counter cation of TFSI^−^ with alternative cations have been reported. TFSI salts with alternative metal cations such as Zn^2+^ and Na^+^, or ammonium ions, have been successfully incorporated into HTL, resulting in enhanced efficiency and stability of PSCs.^[^ ^30^, ^31^ ^]^ Additionally, ionic liquid (IL) dopants containing bulky organic cations have also been considered as alternatives. 1‐butyl‐3‐methylpyridinium bis(trifluoromethylsulfonyl)imide (BMPy), poly(1‐butyl‐3‐vinylimidazolium bis(trifluoromethylsulfonyl)imide) (PVBI), and N‐butyl‐N’‐(4‐pyridylheptyl)imidazolium (BuPyIm) were employed as cations for ionic liquid dopants.^[^ ^32^, ^33^, ^34^ ^]^ Recently, Zhu et al. reported that introducing 1‐butyl‐1‐methylpiperidinium bis(trifluoromethyl sulfonyl)imide (BMPIPTFSI) as a dopant for spiro‐OMeTAD led to the PCE of 23.01%.^[^ ^35^ ^]^

Although previous studies showed advantages of IL additives including low volatility, hydrophobicity, and thermal stability, there is still room for multifunctionality in molecular design of cations. As alkylammonium ions of the additive in spiro‐OMeTAD passivate surface defects on the perovskite surface, cations of ILs act as surface passivators by introducing binding sites that can chemically interact with lead or iodine ions on the perovskite surface.^[^ ^36^, ^37^ ^]^

In this study, the new ionic liquid dopant, bis(trifluoromethylsulfonyl)imide (MMPyTFSI), was applied for spiro‐OMeTAD HTL in PSCs. MMPyTFSI was chosen due to its wide electrochemical window, which is known to provide high chemical stability, making it a promising candidate for improving the long‐term stability of PSCs. Pyrrolidinium and methoxy groups in MMPy ions can interact with iodine and lead sites on the surface of the perovskite, respectively, leading to additional defect passivation effects, while TFSI ions facilitate p‐type doping. Spiro‐OMeTAD doped with MMPyTFSI demonstrated good miscibility and hydrophobicity without additional solvents, resulting in a stable and uniform HTL. The optimized PSC achieved a peak PCE of 23.10% and demonstrated enhanced long‐term stability. The devices exhibited T_80_ values of 1100 h in a humid atmosphere (50% RH, 25 °C, unencapsulated), 1400 h at high temperature (65 °C, dark, encapsulated), and 1100 h under simulated 1 sun illumination (25 °C in ambient air, encapsulated), indicating enhanced moisture, thermal, and photostability, respectively.

## 2 Results and Discussion

### 2.1 Dual Functional Additive for Spiro‐OMeTAD Doping and Surface Passivation of Perovskite

**Figure 1:**
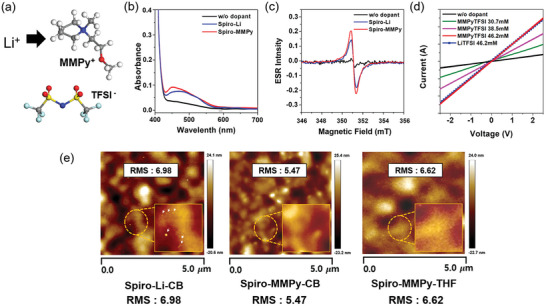
a) Molecular structures of MMPy^+^ and TFSI^−.^ b) Absorbance spectrum for spiro‐OMeTAD films without dopant, with LiTFSI, and with MMPyTFSI. c) ESR Spectrum for spiro‐OMeTAD films without dopant, with LiTFSI, and with MMPyTFSI. d) Current–voltage measurements for undoped; MMPyTFSI‐doped with concentrations of 30.7 mm, 38.5 mm, and 46.2 mm; and LiTFSI doped with the concentration of 46.2 mm. e) AFM images for spiro‐OMeTAD films with LiTFSI from CB‐based solution and with MMPyTFSI from CB‐ and THF‐based solutions.

MMPyTFSI (**Figure** **1**a) exhibits superior solubility in spiro‐OMeTAD solution based on chlorobenzene (CB) compared to LiTFSI, eliminating the need for additional solvents like acetonitrile and tBP, which can degrade the perovskite.^[^ ^29^, ^38^ ^]^ This enhanced solubility is attributed to the weaker cation‐anion ionic bonding in MMPyTFSI, which facilitates its dissolution in less polar antisolvents for perovskite. We also confirmed that this ionic liquid itself does not cause degradation of (FAPbI_3_)_0.95_(MAPbBr_3_)_0.05_ perovskite films when directly contacted with them (Figure S1, Supporting Information).

MMPyTFSI offers a promising solution to the environmental toxicity of CB, a common solvent for spiro‐OMeTAD. By enabling the dissolution of both spiro‐OMeTAD and MMPyTFSI without using CB, MMPyTFSI contributes to a more sustainable fabrication process suitable for mass production.^[^ ^39^, ^40^, ^41^, ^42^, ^43^ ^]^ Among various candidate solvents, both spiro‐OMeTAD and MMPyTFSI showed good solubility in tetrahydrofuran (THF) and dichloromethane (Figure S2, Supporting Information). At a dopant concentration of 46.2 mm, MMPyTFSI demonstrated miscibility in both THF and dichloromethane solutions, while LiTFSI exhibited miscibility only in THF. Comparing the film morphology of spin‐coated spiro‐OMeTAD with the dopants, some aggregates were found in the films with MMPyTFSI in dichloromethane and LiTFSI in THF, whereas a smooth morphology was observed in the film with MMPyTFSI in THF. Based on the miscibility and film morphologies, we chose THF as an eco‐friendly solvent for MMPyTFSI‐doped spiro‐OMeTAD.

The doping effects of MMPyTFSI were investigated with changes in optical and electrical properties of MMPyTFSI‐incorporated spiro‐OMeTAD films. Figure 1b presents ultraviolet–visible (UV–Vis) absorption spectra of spiro‐OMeTAD films on glass substrates in three conditions: undoped, doped with LiTFSI (spiro‐Li), and doped with MMPyTFSI (spiro‐MMPy). Both spiro‐Li and spiro‐MMPy exhibit an absorption band centered at approximately 530 nm, indicative of oxidized spiro‐OMeTAD⁺.^[^ ^44^, ^45^, ^46^ ^]^ Notably, the intensity of this absorption peak is comparable for both doped samples. In contrast, undoped spiro‐OMeTAD shows an absorption band only at 380 nm attributed to the neutral spiro‐OMeTAD species. The oxidation of spiro‐OMeTAD is also detected by electron spin resonance spectroscopy (ESR) (Figure 1c). Both spiro‐Li and spiro‐MMPy films exhibit intense paramagnetic peaks at 350–352 mT, indicating that the oxidation process is triggered by the newly formed spiro‐OMeTAD•^+^TFSI^−^ radicals. In contrast, the signal for the undoped spiro‐OMeTAD film is negligible.

The formation of ionized spiro‐OMeTAD⁺ through oxidation enhances hole concentration within the spiro‐OMeTAD layer. The increased electrical conductivity of spiro‐OMeTAD due to the increased hole concentrations was confirmed by current–voltage (*I*–*V*) measurements performed on spiro‐OMeTAD films (Figure 1d). *I*–*V* curves were obtained using ITO/spiro‐OMeTAD/Au device stacks. Increasing the concentration of MMPyTFSI in spiro‐OMeTAD resulted in steeper I‐V curve slopes, indicative of enhanced conductivity. The conductivities of spiro‐MMPy were determined to be 7.64 × 10^−5^, 1.28 × 10^−4^ and 1.98 × 10^−4^ S cm^−1^ for the MMPyTFSI concentration of 30.7 mm, 38.5 mm, and 46.2 mm, respectively. Spiro‐OMeTAD doped with equivalent concentrations (46.2 mm) of MMPyTFSI and LiTFSI exhibited comparable conductivities. This finding suggests that spiro‐MMPy formed from THF possesses charge transfer capabilities equivalent to those of spiro‐Li formed from CB, making MMPyTFSI an effective dopant for spiro‐OMeTAD.

Figure 1e illustrates the film morphology of spiro‐Li formed from a CB‐based solution and spiro‐MMPy films formed from CB‐ and THF‐based solutions, analyzed using atomic force microscopy (AFM). The spiro‐MMPy films show lower root mean square (RMS) roughness (5.47 nm for CB and 6.62 nm for THF) compared to the spiro‐Li film (6.98 nm for CB). Note that a number of white dots are clearly visible on the surface of spiro‐Li film (marked in the magnified image). These white dots are attributed to the aggregation of LiTFSI due to the migration of Li^+^ ions.^[^ ^47^ ^]^ In contrast, the incorporation of MMPyTFSI into spiro‐OMeTAD results in a morphology without phase segregation, avoiding the appearance of these white spots. Although protrusions are visible in the AFM image of spiro‐MMPy‐CB, these appear more prominent due to the enhanced z‐axis scaling of the AFM image. The smoother and uniform films can enhance interfacial electronic contact and improve charge transport.

**Figure 2:**
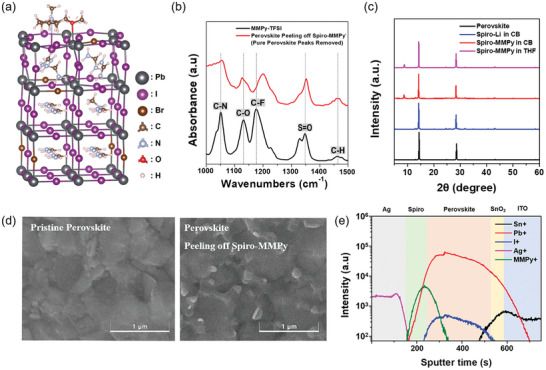
a) The DFT optimized atomic structures of the perovskite (001) surface incorporated with MMPy ions. The Pb, Br, I, C, N, O, and H atoms are indicated by dark gray, brown, purple, light brown, light gray, red, and pink balls, respectively. b) FTIR spectra showing the pure MMPyTFSI peaks and the perovskite peeling off from spiro‐MMPy peaks after subtracting the pure perovskite peaks. c) XRD patterns of perovskite films with spiro‐Li in CB, spiro‐MMPy in CB, spiro‐MMPy in THF, and the pristine perovskite. d) SEM images of the pristine perovskite surface compared with the surface after peeling off spiro‐MMPy. e) ToF‐SIMS depth profile analysis showing the distribution of Sn⁺, Pb⁺, I⁺, Ag⁺, and MMPy⁺ ions across the layers, indicating the depth‐wise ion distribution in the sample structure.

To investigate the chemical interactions between MMPy cations and the spiro‐OMeTAD layer in perovskite films, we conducted first‐principles density functional theory (DFT) calculations. **Figure** **2**a presents the DFT‐optimized atomic structures of the perovskite (001) surface incorporating MMPy cations. The analysis revealed chemical bonds between the lead (Pb) atom of the perovskite surface and the oxygen (O) atom of the MMPy ion, as well as between the nitrogen (N) atom and the iodine (I) atom of the MMPy ion. The Pb─O bond length was calculated to be 3.08 Å, while the N─I bond length was 4.23 Å. The binding energy of an MMPy ion to the perovskite surface was determined to be −3.30 eV. The partial density of states (PDOS) shown in Figure S4 (Supporting Information) indicated that the oxygen of the MMPy ions readily hybridized with the surface Pb atoms, contributing significantly to the valence band density of states of the perovskite. In contrast, the N─I bonds were characterized by their weak strength and long bond length, leading to a localized and distinct density of states that was separated from the perovskite state.

The chemical interactions between the N and O sites of MMPy and the perovskite film were experimentally confirmed using Fourier‐transform infrared spectroscopy (FTIR). Figure 2b presents the FTIR spectra of MMPyTFSI and the perovskite film prepared by coating spiro‐MMPy onto the perovskite and then removing the spiro‐OMeTAD layer with CB. By subtracting the peaks corresponding to pure perovskite from the spectrum of the perovskite film with spiro‐MMPy removed, characteristic absorption bands associated with C─N, C─O, C─F, S═O and C─H functional groups in MMPyTFSI were observed. Comparing the peaks to those of pure MMPyTFSI, the C─O peak was shifted from 1132 to 1126 cm^−1^, and the C─N peak was shifted from 1051 to 1055 cm^−1^. Full spectra of FTIR were demonstrated in Figure S5 (Supporting Information). Furthermore, the chemical interaction was also supported by X‐ray photoelectron spectroscopy (XPS) analysis (Figure S7, Supporting Information). A blue shift of the peaks for Pb4f_5/2_, Pb4f_7/2_, I3d_3/2_, and I3d_5/2_ was observed in the perovskite film with spiro‐MMPy removed compared to the peaks from a pristine perovskite film. In the C1s spectra, a peak from pyrrolidinium was detected at 286.4 eV.

Figure 2c presents the X‐ray diffraction (XRD) patterns for a pristine perovskite film; a perovskite film covered with a spiro‐Li layer from a CB solvent, and perovskite films covered with spiro‐MMPy layers from both CB and THF solvents. A distinct peak appears at 8.7° in the XRD patterns of the spiro‐MMPy samples, which is absent in the undoped and LiTFSI‐doped sample. As Rao et al. reported that the incorporation of pyrrolidinium cations into PbI₂ alters the crystallization pathway, leading to the formation of the perovskite structure.^[^ ^48^ ^]^ Similarly, the 8.7° peak observed in our study aligns with the peak in Figure S6 (Supporting Information), representing the XRD pattern of the PbI₂‐MMPyTFSI reacted powder. It indicates the chemical interaction between PbI₂ in the perovskite and MMPy. Indeed, the new phase was observed in the scanning electron microscopy (SEM) image of the surface of the perovskite film after the removal of the spin‐coated spiro‐MMPy layer using CB solvent (Figure 2d). The SEM images also show that this new phase is predominantly formed around the grain boundaries of the perovskite. The presence of this secondary phase suggests that MMPyTFSI in a spiro‐OMeTAD layer acts as a surface treatment agent, similar to alkylammonium halides, that chemically reacts with the perovskite to passivate surface defects.

We employed time‐of‐flight secondary ion mass spectrometry (ToF‐SIMS) to analyze the spatial distribution of the MMPyTFSI dopant within the perovskite and spiro‐OMeTAD stack. The results revealed that MMPy ions are concentrated at the interface between the perovskite and spiro‐OMeTAD (Figure 2e). In contrast, in the sample of spiro‐Li, Li ions cations were evenly distributed throughout the spiro‐OMeTAD layer and appeared to have penetrated into the perovskite layer (Figure S8, Supporting Information). This observation is consistent with the previous result,^[^ ^31^, ^49^ ^]^ which identified the detrimental effects of migrated Li ions on the phase stability of perovskite. The distribution of MMPy suggests that the MMPy contained in spiro‐OMeTAD readily undergoes a chemical reaction with the perovskite at the interface.

**Figure 3:**
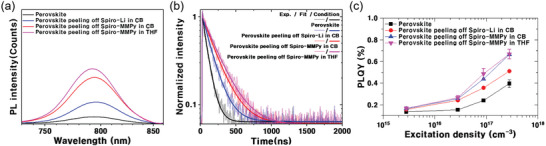
a) Steady‐state photoluminescence (PL) spectra. b) Time‐resolved photoluminescence (TRPL) decay, and c) photoluminescence quantum yield (PLQY) under various excitation densities for perovskite films of pristine, peeling off a LiTFSI‐doped spiro‐OMeTAD layer, peeling off a MMPyTFSI‐doped spiro‐OMeTAD layer in CB, and peeling off a MMPyTFSI‐doped spiro‐OMeTAD layer in THF.

To assess surface passivation effects of MMPyTFSI in spiro‐OMeTAD films as a dopant, photoluminescence (PL) analyses were conducted on perovskite films after removing spiro‐Li layers and spiro‐MMPy layers. Steady‐state PL spectra revealed that the perovskite film after removing spiro‐MMPy exhibits significantly higher PL intensity than a pristine perovskite film (**Figure** **3**a). Spiro‐Li also enhanced PL intensity from the perovskite, but the increase was lower than that observed with spiro‐MMPy.

To enable a more quantitative comparison of charge recombination, time‐resolved PL (TRPL) decay spectra were measured under low‐intensity pulsed excitation, revealing a mono exponential decay characteristic of nonradiative‐trap‐induced recombination (Figure 3b). The perovskite reacted with MMPyTFSI exhibited a longer carrier lifetime of approximately 187.21 ns, which is 2.4 times longer than that of the pristine perovskite layer (77.48 ns). Additionally, the perovskite peeling off spiro‐MMPy in THF showed a carrier lifetime of 193.67 ns. The higher steady‐state photoluminescence (SSPL) intensity and the prolonged early‐stage time constant of the TRPL decay indicate the reduction of nonradiative recombination paths through surface treatments that passivate defect sites on the perovskite surface and grain boundaries.

To further investigate the effects of surface passivation by MMPy, photoluminescence quantum yield (PLQY) was measured. Figure 3c presents the PLQY of perovskite films as a function of photo‐excitation density. The PLQY from the spiro‐MMPy‐removed perovskite is consistently higher than those from the pristine perovskite and the spiro‐Li‐removed perovskite across all excitation densities, indicating that MMPyTFSI treatment significantly enhances the radiative efficiency of the perovskite layer. The PL and PLQY results suggest that MMPyTFSI in spiro‐OMeTAD is effective in passivating the perovskite surface, thereby enhancing the overall photoelectronic performance of the perovskite material.

### 2.2 Enhanced Photovoltaic Performance

**Figure 4:**
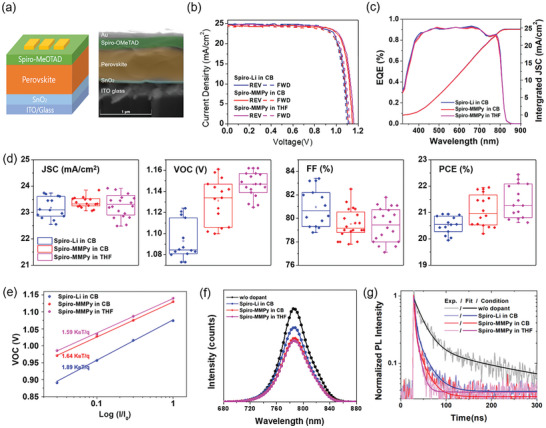
a) Schematic structure and cross‐sectional SEM image of PSCs. b) Current density–voltage (*J*–*V*) curves for the devices with spiro‐Li in CB, spiro‐MMPy in CB, and spiro‐MMPy in THF, demonstrating the photovoltaic performance under forward (FWD) and reverse (REV) scan directions. c) EQE spectra and curves for integrated short circuit current density. d) Box plots of *V*_OC_, *J*_SC_, FF, and PCE for the devices. e) *V*_OC_ versus light intensity curves for the devices, showing ideality factors (*n*) for each HTL condition. f) Steady‐state photoluminescence (SSPL) spectra for the perovskite films with different HTL conditions. g) TRPL decay curves comparing the charge carrier lifetimes under different HTL conditions.

The consistent results of MMPyTFSI's superior doping performance and surface passivation effectiveness encourage further investigation into its potential applications in photovoltaic devices. We fabricated PSCs of a n–i–p structure with an ITO substrate/SnO₂ nanoparticles/(FAPbI_3_)_0.95_(MAPbI_3_)_0.05_/spiro‐OMeTAD /Au electrode and evaluated photovoltaic performances (**Figure** **4**a). To compare the effects of different dopants and solvents, we fabricated PSCs using spiro‐OMeTAD doped with LiTFSI dissolved in CB, MMPyTFSI dissolved in CB or THF. Optimizing the solvent conditions by transitioning from CB to THF significantly reduced the consumption of spiro‐OMeTAD (Figure S9 and Table S3, Supporting Information). Specifically, the usage of spiro‐OMeTAD decreased from 90.9 mg mL^−1^ with CB to 70 mg mL^−1^ with THF, representing a reduction of approximately 22.99%. This modification directly contributed to lower material costs and enhanced resource efficiency in the fabrication process.

Figure 4b,d shows the current density–voltage (*J*–*V*) curves under AM 1.5G‐1 sun illumination and statistical results for photovoltaic performance metrics, respectively, of these devices. PSCs employing spiro‐MMPy consistently exhibited higher power conversion efficiencies (PCEs) than those using spiro‐Li, regardless of the solvent. Statistical analysis revealed that the increased PCE of spiro‐MMPy devices was primarily due to a higher open‐circuit voltage (*V*_OC_) with minimal impact on short‐circuit current density (*J*_SC_) and fill factor (FF). The best‐performing device, using spiro‐MMPy in THF, achieved 23.10%, *V*_OC_ of 1.16 V, *J*_SC_ of 24.68 mA cm⁻^2^, and FF of 80.7%. The device with spiro‐Li in CB reached a PCE of 22.35%, *V*_OC_ of 1.11 V, *J*_SC_ of 24.80 mA cm⁻^2^, and an FF of 81.2%. Table S4 (Supporting Information) provides a comprehensive summary of device performance and statistical data. The large improvement in *V*_OC_ can be attribute to the suppression of nonradiative recombination at the spiro‐OMeTAD‐perovskite interface.

Figure 4c depicts the external quantum efficiency (EQE) curves for the devices, demonstrating excellent agreement between the integrated *J*_SC_ values from EQE and measured *J*_SC_ from *J*–*V* curves. The device with spiro‐MMPy and spiro‐Li achieved the *J*_SC_ values of 24.64 mA cm⁻^2^ and 24.67 mA cm⁻^2^, respectively. A subtle enhancement in EQE was observed near 780 nm for the PSC with spiro‐MMPy in CB, but this had negligible on *J*_SC_. The results of maximum power point tracking (MPPT) for these devices under AM 1.5G‐1 sun illumination in ambient air are shown in Figure S10 (Supporting Information). The stabilized PCE of the device using MMPyTFSI in THF solvent was 22.3%, while the that of the device using LiTFSI in CB solvent was 21.8%. Notably, the output power remained very stable during the 100 s MPPT measurement.

To elucidate the underlying mechanisms contributing to the enhanced PCE of PSCs with spiro‐MMPy, ideality factors were investigated. The ideality factor (*n*) was extracted by fitting the slopes of the V_OC_ versus light intensity plot, *nk*_B_ *T*/*q*, where *k*_B_ is the Boltzmann constant, *T* temperature, and *q* elementary charge, suggesting the junction quality and the type of recombination. The ideality factors for spiro‐Li in CB, spiro‐MMPy in THF, and spiro‐MMPy in CB were determined to be 1.89, 1.59, and 1.64, respectively (Figure 4e). The ideality factor value closest to 1 observed for spiro‐MMPy in THF indicates the lowest trap‐assisted recombination rate,^[^ ^50^ ^]^ this correlation with improved V_OC_ values in PSCs incorporating spiro‐MMPy further supports the beneficial role of MMPyTFSI in suppressing non‐radiative recombination. As Jiang et al. reported that processing spiro‐OMeTAD in THF increases crystallinity and enhances hole mobility.^[^ ^51^ ^]^

For PL analysis to compare charge transport efficiency from the perovskite to spiro‐OMeTAD, a glass / perovskite / spiro‐OMeTAD structure was employed. SSPL spectra revealed, a decrease in PL intensity upon the introduction of a spiro‐OMeTAD layer, which indicates that hole extraction occurred by the HTL (Figure 4f). The spiro‐MMPy in CB sample exhibits a lower PL intensity compared to the spiro‐Li in CB sample, with the spiro‐MMPy in THF sample showing an even greater reduction in PL intensity. TRPL analysis corroborated these findings, showing faster PL decay kinetics for spiro‐MMPy‐based samples (Figure 4g). The carrier lifetimes calculated using a biexponential decay model. PL lifetimes were 1.35 ± 0.11 ns and 1.26 ± 0.17 ns for the spiro‐MMPy samples prepared in CB and THF, respectively, and 3.53±0.45 ns for the spiro‐Li samples in CB (Table S5, Supporting Information). The shorter lifetimes observed in spiro‐MMPy‐based samples doped with MMPyTFSI suggest more efficient hole transport from the perovskite to spiro‐OMeTAD compared to spiro‐Li samples. Therefore, the role THF as the solvent attributed to enhance the hole extraction and transport efficiency of spiro‐MMPy. Ultraviolet photoelectron spectroscopy (UPS) measurements revealed a more favorable band alignment between the perovskite and spiro‐OMeTAD, suggesting that the HOMO level of the spiro‐MMPy in CB is better suited for hole extraction from the perovskite than spiro‐Li in CB (Figure S11, Supporting Information). Noticeably, the spiro‐OMeTAD with MMPyTFSI processed by THF demonstrates a more favorable HOMO level for hole extraction pathway.

The observed performance enhancement in PSCs incorporating MMPyTFSI doping is primarily attributed to the beneficial role of MMPyTFSI as the dopant, which plays a crucial role in improving device efficiency. Additionally, the use of THF as the solvent contributes to enhanced solubility and reduced material consumption, the significant increase in power conversion efficiency (PCE) is primarily due to the MMPyTFSI dopant's ability to enhance hole transport and passivate surface defects on the perovskite layer. This enhanced charge transport has a greater impact than the minor improvements from solvent changes. This reinforces the conclusion that the dopant is the key factor responsible for the observed improvements in photovoltaic performance.

**Figure 5:**
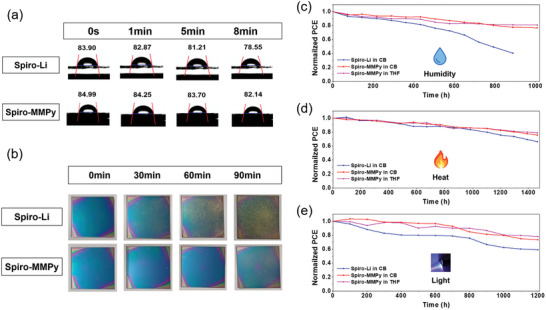
a) Contact angle measurements of spiro‐OMeTAD films doped with LiTFSI and MMPyTFSI. b) Visual stability of perovskite thin films (spiro‐Li and spiro‐MMPy) under humid Air Exposure. c) Stability test in a humid atmosphere (50% relative humidity, 25 °C) for 1000 h without encapsulation. d) Thermal stability test 65 °C for 1500 h with encapsulation. e) MPPT (maximum power point tracking) under 1 sun illumination in ambient air at 25 °C for 1200 h (50 d) with encapsulation.

Given the hydrophobic nature of MMPy ions, PSCs with spiro‐MMPy demonstrated enhanced stability against moisture. Contact angle measurements revealed a higher contact angle for spiro‐MMPy compared to spiro‐Li, indicating a more hydrophobic surface of HTL (**Figure** **5**a). Furthermore, high contact angle was retained over time on the spiro‐MMPy. Shelf‐life tests of perovskite/spiro‐OMeTAD bilayer samples under high humidity conditions (room temperature (RT), and ≈80% relative humidity (RH)) further corroborated this behavior. While the surface of the spiro‐Li sample exhibited a color change from green‐yellow to yellow after 90 minutes, the spiro‐MMPy sample remained stable with negligible color changes (Figure 5b). XRD analysis revealed a significant increase in the PbI₂ peak at 12.6° for the spiro‐Li sample after exposure to humid air, indicating severe degradation of the perovskite (Figure S12, Supporting Information). In contrast, the spiro‐MMPy sample showed only a minor PbI₂ diffraction peak. In the long‐term stability test of unencapsulated PSCs under humid air (RT, ≈50% RH), the *T*_80_ values of a PSC with spiro‐Li prepared in CB was reached 450 h, while the PSCs with spiro‐MMPy in THF and CB exhibited notably improved humidity stability with *T*_80_ values of 1100 h in THF and 800 h in CB.

The thermal stability of the PSCs at 65 °C in the dark was also enhanced by the use of spiro‐MMPy. Encapsulated devices with the spiro‐MMPy prepared in THF and CB exhibited T_80_ values of 1400 and 1300 h, respectively, surpassing the 1100 hours from spiro‐Li‐based devices in CB (Figure 5d). Under continuous light exposure of simulated 1 sun illumination at 25 °C in ambient air, encapsulated PSCs with the spiro‐MMPy prepared in THF and CB demonstrated T_80_ values of 1100 and 1000 h, respectively, while those with spiro‐Li in CB reached only 500 h (Figure 5e). These results show that more hydrophobic properties and defect passivation effects of spiro‐MMPy not only improves humidity resistance but also enhances thermal, and light stability, making it a more suitable candidate for developing long‐lasting, high‐performance perovskite solar cells.

## 3 Conclusion

We have developed and evaluated a novel ionic liquid dopant, MMPyTFSI, as a promising alternative to the conventional dopant, LiTFSI, for use in spiro‐OMeTAD in PSCs. MMPyTFSI effectively addresses the limitations associated with LiTFSI, including hygroscopicity, lithium‐ion migration, and the need for toxic and additional solvents. By inducing efficient p‐type doping of the HTL and simultaneously passivating the perovskite surface through a chemical reaction with MMPy, MMPyTFSI facilitates efficient hole transport and suppresses non‐radiative recombination at the perovskite‐HTL interface. These synergistic effects lead to enhanced PCE and improved long‐term stability in PSCs incorporating MMPyTFSI‐doped spiro‐OMeTAD. PSCs utilizing MMPyTFSI showed a substantial increase in PCE from 22.35% to 23.10%. Moreover, the results of long‐term stability tests highlight the superior performance of spiro‐MMPy in terms of humidity resistance, thermal stability, and light stability. We confirmed that the device operated stably without any performance degradation when MMPyTFSI‐doped spiro‐OMeTAD was used in the environmentally friendly solvent tetrahydrofuran instead of chlorobenzene. Additionally, reducing the amount of spiro‐OMeTAD was found to be more effective when using THF. These findings underscore the potential of MMPyTFSI as a promising HTL dopant for the development of efficient and durable PSCs and provide valuable insights for the future design and commercialization of perovskite‐based renewable energy solutions.

## 4 Experimental Section

### Materials

Pre‐patterned ITO glass substrates were purchased from Biotain Crystal, and the FTO glass substrates were purchased from Pilkington. SnO_2_ was purchased from Alfa Aesar. N,N‐dimethylformamide (DMF, 99.8%), dimethyl sulfoxide (DMSO, 99.7%), chlorobenzene (CB,99.8%), tetrahydrofuran (THF, 99.9%), formamidinium iodide (FAI, 98%), methylammonium bromide (MABr, 98%), and isopropanol (IPA) were purchased from Sigma‐Aldrich. Lead (ІІ) iodide (PbI_2_, 99%), lead bromide (PbBr_2_, 99%), 2‐bromoethyl methyl ether, 1‐methylpyrrolidine (99%), and lithium bis(trifluoromethanesulfonyl)imide were purchased from Tokyo Chemical Industry. spiro‐OMeTAD was purchased from Luminescence Technology Corp.

### Synthesis of MMPy‐TFSI

An amount of (1.34 g 4.7 mmol) lithium bis(trifluoromethane sulfonyl) imide salt was dissolved in 15 mL distilled water. In 15 mL distilled water, (1.0 g 4.5 mmol) MMPy‐Br was dissolved. The two aqueous solutions were mixed together and then stirred at room temperature for 3 h. The product (organic phase) was separated from the aqueous phase by a separating funnel and was washed with distilled water twice to remove any water‐soluble impurities. Yield: 85%. ^1^H NMR(400 MHz, CDCl_3_) δ (ppm): 2.21‐2.28 (m, 4H), 3.10 (s, 3H), 3.38 (s, 3H), 3.53‐3.62 (m, 6H), 3.76‐3.80(m, 2H). ^13^C NMR (101 MHz, CDCl_3_) *δ* = 124.75, 121.56, 118.37, 115.17, 66.46, 65.80, 65.77, 65.75, 63.60, 63.57, 63.54, 59.16, 49.09, 49.05, 49.01, 21.49, 21.39 (Figure S14, Supporting Information).

### Solar Cell Fabrication

Perovskite solar cells were fabricated with a configuration of ITO or FTO/SnO_2_ nanoparticle/perovskite/spiro‐OMeTAD /Au. ITO or FTO glass substrates were cleaned by sonicating them in acetone and isopropanol (IPA). The substrates were treated with UV‐ozone surface cleaner for 15 min. SnO_2_ electron transport layers (ETLs) were prepared by spin‐coating a solution of SnO_2_ nanoparticles, dissolved in deionized water at a 1:5 ratio, on an ITO substrate at a spin rate of 3000 rpm for 30 s; this was followed by annealing on a hotplate in air at 100 °C for 10 min. The substrates were treated with UV‐ozone surface cleaner for 15 min. The perovskite precursor was prepared by dissolving 582 mg PbI_2_, 217 mg FAI, 2mg PbBr_2_, 25 mg MACl, and 7.4 mg MABr in 0.8 mL N,N‐dimethylformamide(DMF) and 0.1 mL dimethyl sulfoxide (DMSO) and filtering with PTFE filter. The dissolved solution was then spin‐coated on ITO/SnO_2_ substrate at 500 rpm for 5 s, then for 8 s at 1200 rpm, and then for 12 s at 5000 rpm. During the last coating step, 1 mL diethyl ether was dropped on the spinning film. Then, the films were annealed at 100 °C for 1 h. After annealing and cooling to room temperature. Then 100 mg of spiro‐OMeTAD in 1.1 mL of chlorobenzene, followed by doping with 23 µL of LiTFSI (54 mg in 1mL acetonitrile) or 23 µL of MMPyTFSI (43.8 mg in 1 mL acetonitrile) and 39 µL of 4‐tBP. Additionally, 70 mg of spiro‐OMeTAD was dissolved in 1 mL of tetrahydrofuran, followed by doping with 19.6 µL of MMPyTFSI (without acetonitrile) and 39 µL of 4‐tBP. The solutions were filtered using a PTFE filter after preparation and then spin‐coated at 3000 rpm for 30 s on the spinning film. Finally, 80 nm of gold electrode was thermally evaporated on top of the HTL to complete the device. The active area of the cells was 0.094 cm^2^

### Characterization

UV–vis was performed using an OPTIZEN system. Electron spin resonance (ESR) analysis was carried out using the EMXplus‐9.5/12/P/L System. Conductivity was measured using a Zennium‐Pro (Zahner). The surface morphology and chemical composition of the samples were analyzed using an AFM‐Raman spectrometer (INNOVA‐LABRAM HR800, Horiba/Bruker). The chemical composition of the samples was analyzed using a Fourier transform infrared spectrometer (FTIR, Bruker AlPha II). To identify the surface orientation of the perovskite, an X‐ray diffractometer (XRD, Bruker, D2 Phaser A26‐X1‐A2G0B2A0) was used. The morphology of the perovskite was investigated using a field emission scanning electron microscope (FE‐SEM, TESCAN CLARA). X‐ray photoelectron spectroscopy (XPS) and ultraviolet photoelectron spectroscopy (UPS) analyses were performed using an X‐ray & UV photoelectron spectrometer (K‐alpha+, Thermo). Time of flight secondary ion mass spectrometry (TOF‐SIMS) was conducted to obtain depth profiling (TOF.SIMS 5, ION‐TOF GmbH). To prepare the perovskite films for PL, TRPL, and PLQY measurements, samples with a glass/perovskite/spiro‐OMeTAD structure using LiTFSI, MMPyTFSI, and without dopant were fabricated. The perovskite and spiro‐OMeTAD layers were prepared following the procedure detailed in Section 4.3 on solar cell fabrication. For sample preparation, the films in a chlorobenzene (CB) solution were dipped for 5 s to remove the spiro‐OMeTAD layer. To ensure complete removal of residual solvent, the samples were then dried in an 80 °C oven for 15 min. For the photoluminescence (PL) and time‐resolved photoluminescence (TRPL) spectroscopy were employed a time‐correlated single‐photon counting module (TCSPC FluoTime 300 by Picoquant, GmbH). The film was measured by LDH‐P‐C‐520 (Picoquant 520 nm pulse has 50 ps FWHM) laser source, PDL 820 (Picoquant) laser driver, and UV‐red‐PMT monochrometer (MSH300PQ‐0002, Picoquant). The PL measured 20 MHz frequency, 1.42 µJ/cm^2^/pulse energy density and the TRPL also measured 20 MHz frequency, 1.42 µJ/cm^2^/pulse. For the photoluminescence quantum yield (PLQY) was measured using a integration sphere (newport, 819C‐IC‐3.3). They were measured by QEPro (Ocean insight, 350–950 nm). For excitation, continuous 532 nm laser (lasercentury, GL532N3‐200) source was employed. PLQY 5.3 µW/cm^2^/s energy density was measured and OD filter was employed to diverse laser intensity. The *J*−*V* curves were measured with a forward scan from −0.2 to 1.5 V or a reverse scan from 1.5 to −0.2 V. The photovoltaic characteristics were examined using a solar simulator equipped with a xenon lamp and a source meter (Keithley 2450 Instruments) at AM 1.5 G 100 mW cm^−2^. All of the devices were measured by masking the active area of 0.0804 cm^2^ using a metal mask. Maximum power point tracking (MPPT) measurements were performed using a K3600 solar cell reliability system (McScience) with LED‐array illumination emitted at 100 mW cm^−2^ in AM 1.5G simulations. External quantum efficiency (EQE) was measured using a quantum efficiency measurement tool (Model QuantX, Newport) at room temperature in air.

### DFT Calculation

First‐principles density functional theory (DFT) calculations using Vienna ab initio simulation package (VASP) were performed.^[^ ^52^ ^]^ Kohn‐Sham wave functions are expanded by a series of plane waves with a kinetic energy cut‐off of 400 eV. The projected augmented wave (PAW) potentials^[^ ^53^ ^]^ and exchange‐correlation functional of Perdew‐Burke‐Ernzerhof (PBE)^[^ ^54^ ^]^ were used for electron‐ion and electron–electron interactions, respectively. The convergence criterion of the total energy is 10^−4^ eV. For structural optimizations were converged when the Hellmann‐Feynman forces on every ion were less than 0.05 eV Å^−1^. Cubic phase of FA_0.92_MA_0.08_Pb(I_0.93_Br_0.07_)_3_ bulk material with lattice constant as 6.362 Å was used. For the FA_0.92_MA_0.08_Pb(I_0.93_Br_0.07_)_3_ (001) slab model, the (2 × 2) surface unit cell and three perovskite layers were used. The *k*‐point mesh was set to Monkhorst‐Pack scheme with a 2 × 2 × 1. The bottom two perovskite layers are fixed and a first surface layer is fully relaxed. To describe long‐range van der Waals interaction that plays an important role in hybrid perovskites, the DFT‐D2 method was adopted.^[^ ^55^ ^]^

## Conflict of Interest

The authors declare no conflict of interest.

## Supporting information



Supporting Information

## Data Availability

The data that support the findings of this study are available from the corresponding author upon reasonable request.
