# Supplementary material for: A Versatile Ionic Liquid Additive for Perovskite Solar Cells: Surface Modification, Hole Transport Layer Doping, and Green Solvent Processing
Source: Adv Sci (Weinh). 2025 Jan 9;12(8):2412959. doi: 10.1002/advs.202412959 (PMC11848532; doi:10.1002/advs.202412959)
Supplement: Supplementary file 1 — Supporting Information [file ADVS-12-2412959-s001.docx]

Supporting Information

**A Versatile Ionic Liquid Additive for Perovskite Solar Cells: Surface Modification, Hole Transport Layer Doping, and Green Solvent Processing**

Seong-Jin Jeong, Sung Hwan Park, Siwon Yun, Meng Qiang Li, Dasol Kim, Yongchan Kim, Yun Hee Chang, Jaewon Lee*, Jongchul Lim*, Tae-Youl Yang *

**
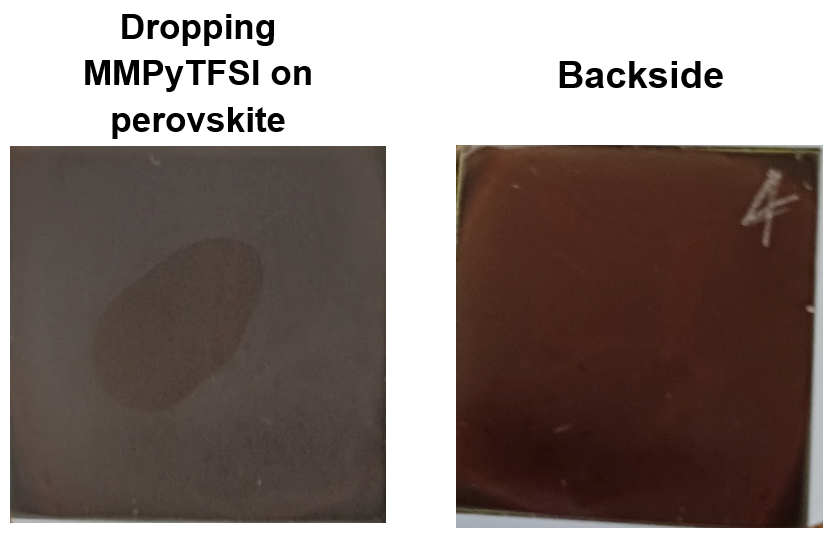
**

**Figure S1.** Photographs of perovskite films from with droplets of MMPyTFSI

**Table S1.** Solubility of Spiro-OMeTAD and MMPyTFSI in Various Solvents and Their Compatibility with Perovskite.

| Solution | Spiro-OMeTAD | Add MMPyTFSI | Compatibility perovskite |
| --- | --- | --- | --- |
| Chlorobenzene | Clear | Not dissolved |  |
| Anisole | Clear | Not dissolved |  |
| Tetrahydrofuran | Clear | Clear | Good |
| Dichloromethane | Clear | Clear | Brinded |
| 1-butyl alcohol | Hazy |  |  |
| Tert butyl alcohol | Hazy |  |  |
| 2-propenal | Hazy |  |  |

**
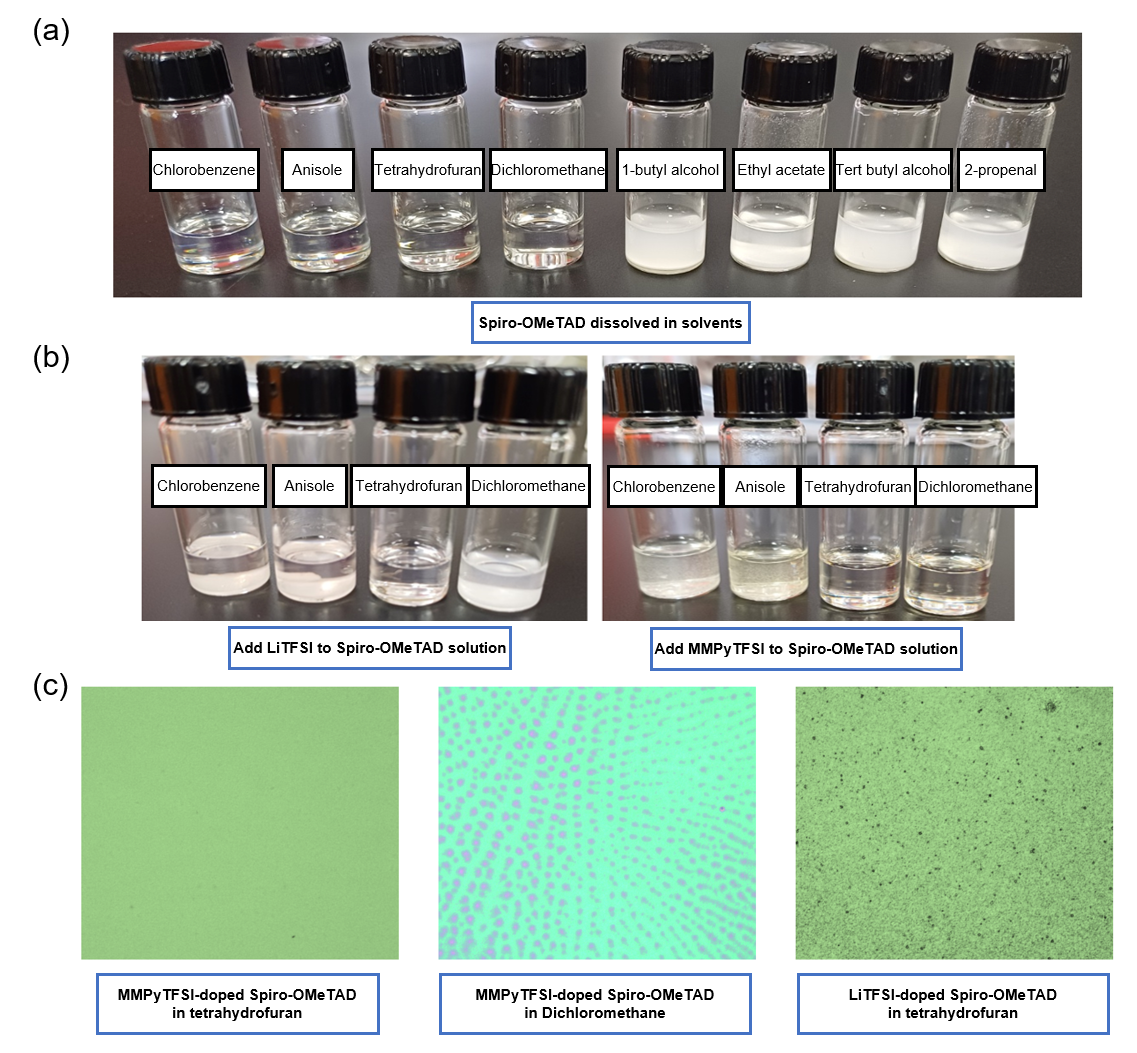
**

**Figure S2.** (a) Photographs of Spiro-OMeTAD dissolved in various solvents, (b) Photographs of solutions prepared by dissolving 46.2 mM of LiTFSI and MMPyTFSI without any other solvent, (c) Optical microscopy (OM) images of Spiro-OMeTAD films made using the dissolved solvents.

**
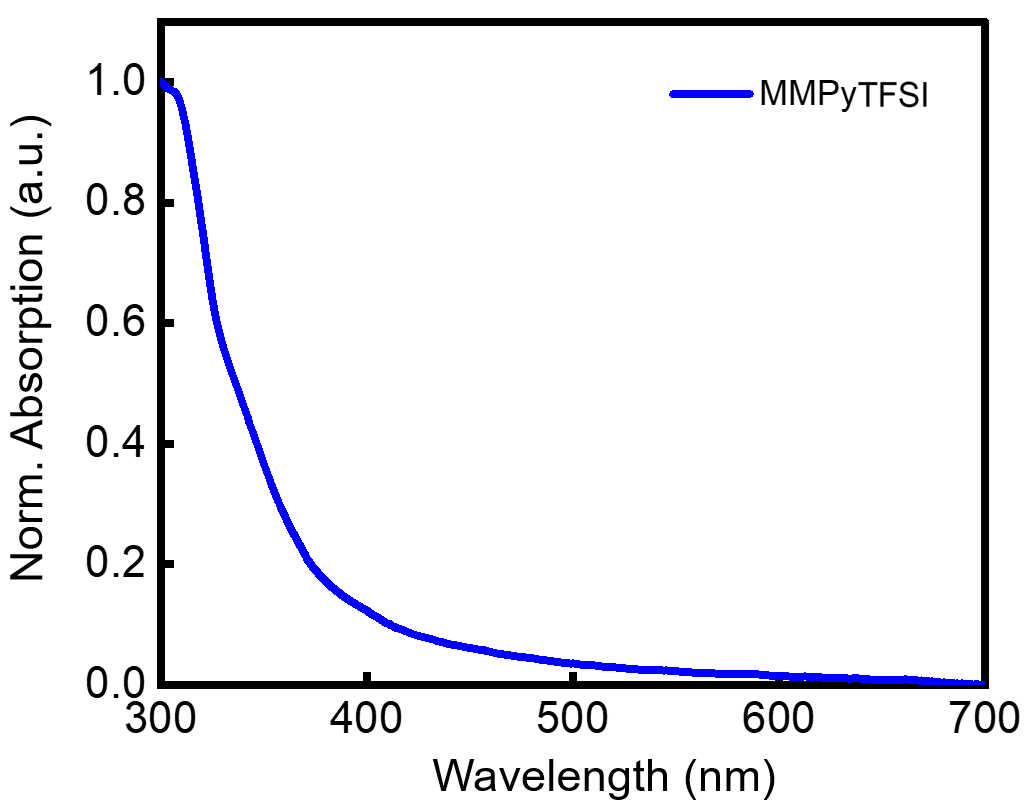
**

**Figure S3**. UV-vis Absorbance spectrum of MMPyTFSI


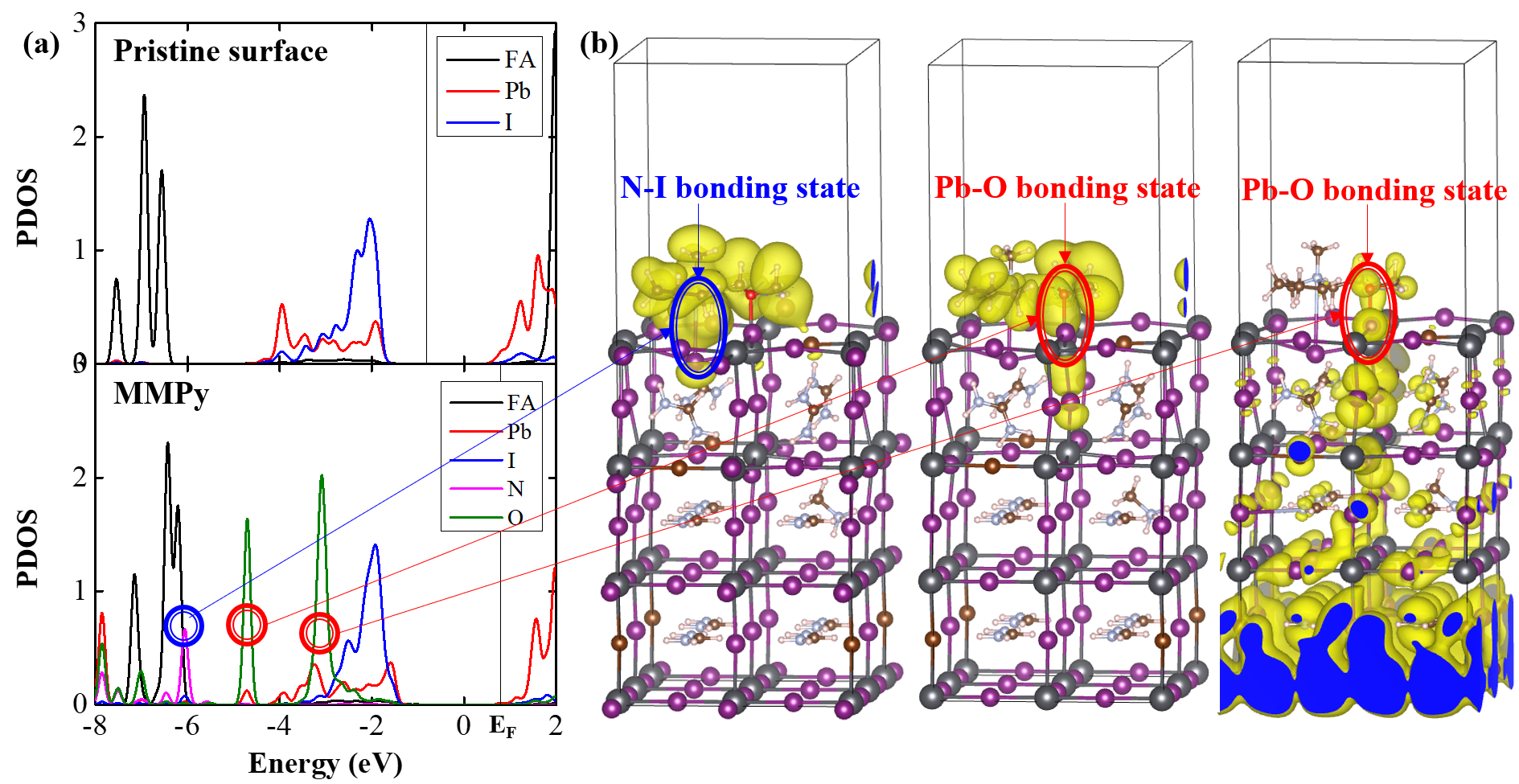


**Figure S4**. (a) Partial density of states (PDOS) of pristine perovskite (001) surface (upper panel) and the perovskite surface with MMPy ions added (bottom panel), (b) Partial charge density corresponding to the localized sharp peaks in PDOS associated with the interaction between MMPy ions and perovskite surfaces.

**
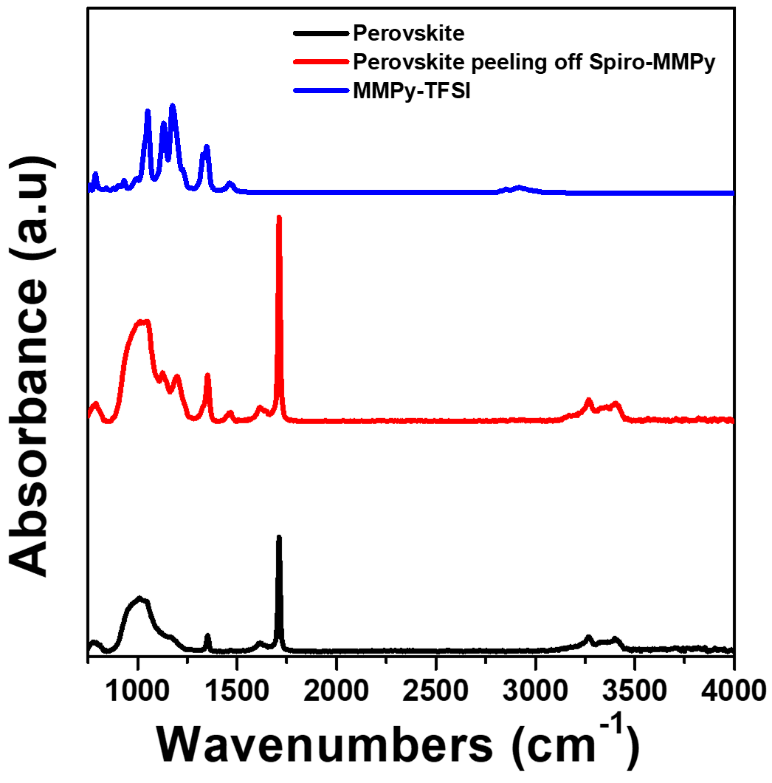
**

**Figure S5.** Full spectra of FTIR analyses.


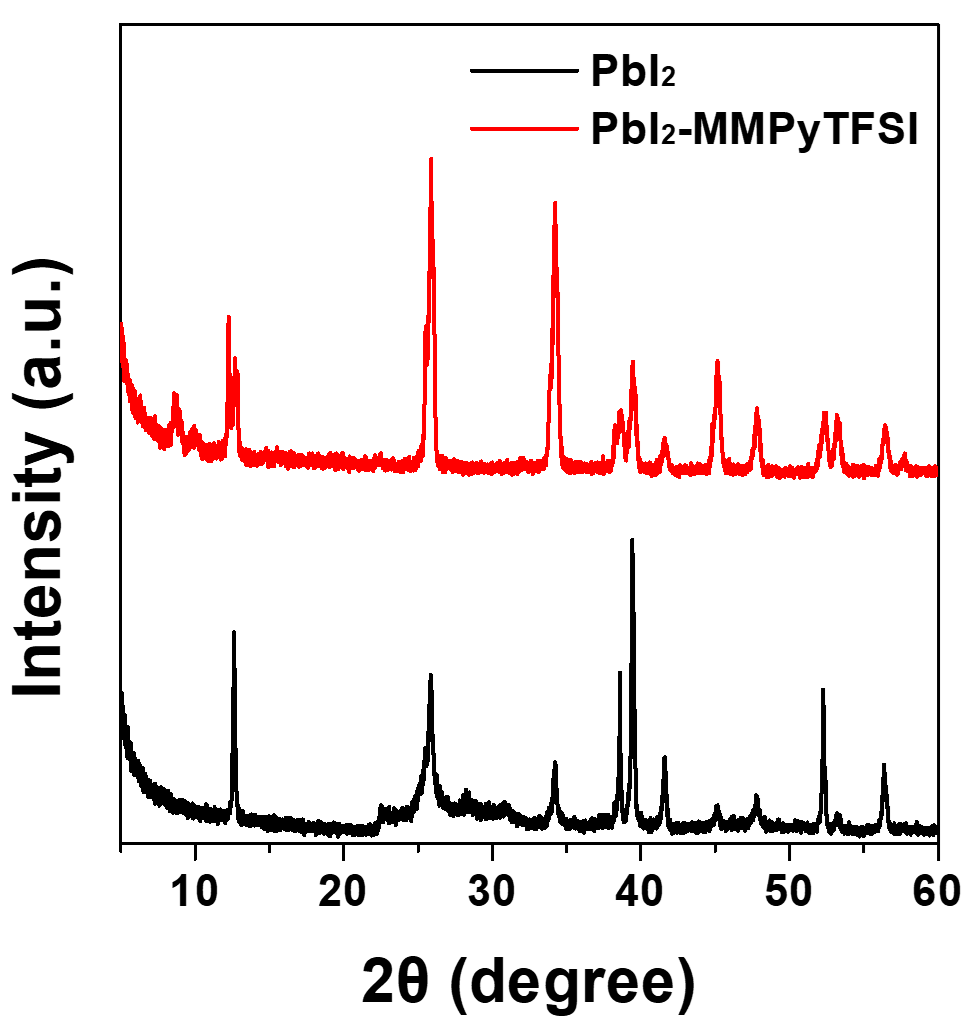


**Figure S6.** XRD Patterns of PbI₂ Powder and PbI₂ Reacted with MMPyTFSI


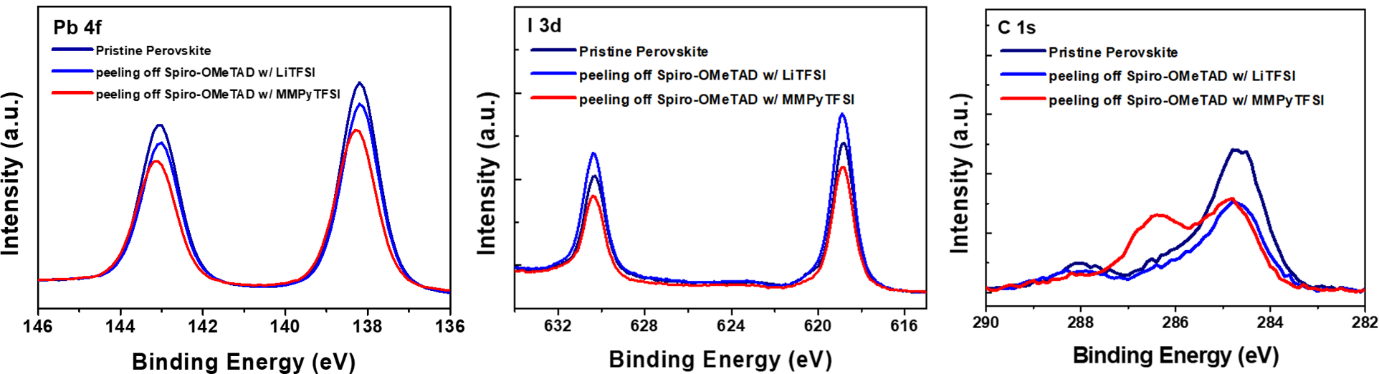


**Figure S7.** X-ray Photoelectron Spectroscopy (XPS) Analysis of Pb 4f, I 3d, and C 1s Binding Energies for Pristine Perovskite, and Perovskite After Peeling Off Spiro-Li and Spiro-MMPy Layers.


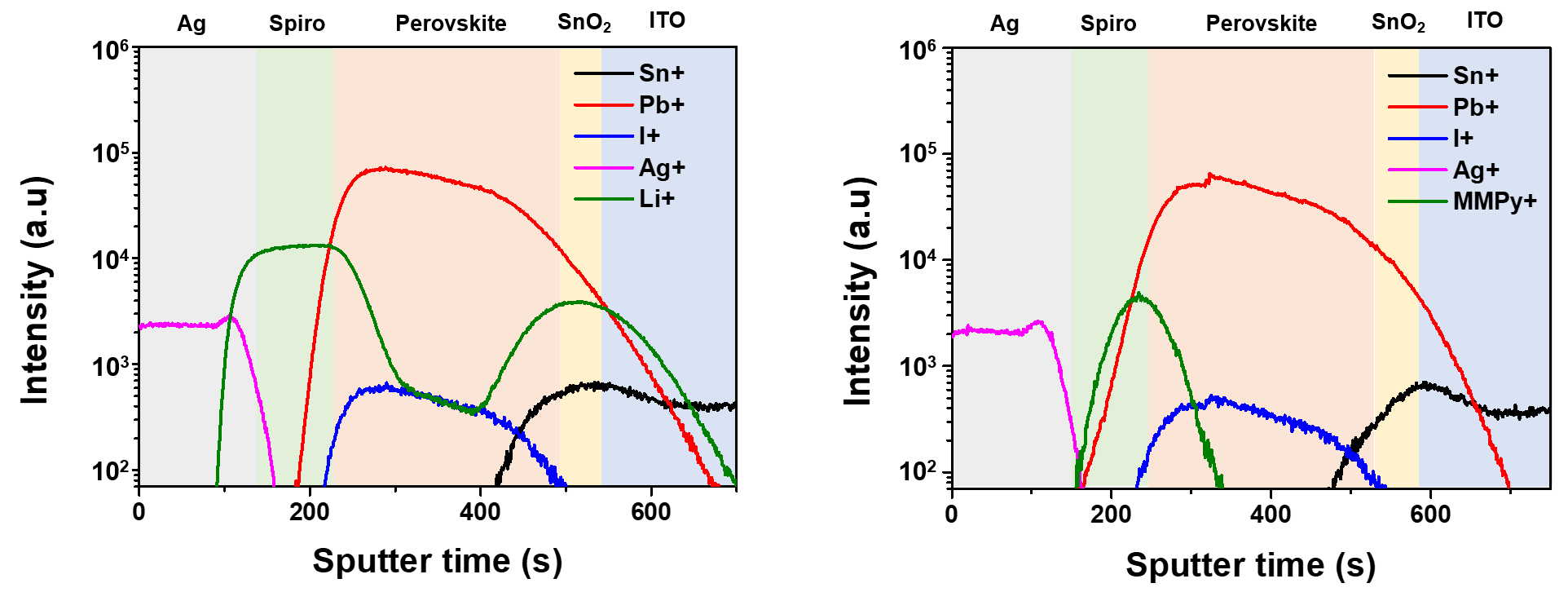


**Figure S8.** Depth Profiling of Elemental Distribution in PSCs Using ToF-SIMS: Comparison of Li^+^ and MMPy^+^ Distribution Across Ag/Spiro-OMeTAD/Perovskite/SnO₂/ITO Layers.

**Table S2.** The Fitted Carrier Lifetimes of Pristine Perovskite and Perovskite Films After Peeling Off Spiro-Li and Spiro-MMPy Layers.

|  | **Tau 1 [ns]** |
| --- | --- |
| Perovskite | 77.48±3.8 |
| Perovskite peeling off Sprio-Li in CB | 120.11±13.45 |
| Perovskite peeling off Spiro-MMPy in CB | 187.21±4.25 |
| Perovskite peeling off Spiro-MMPy in THF | 193.67±11.50 |

**
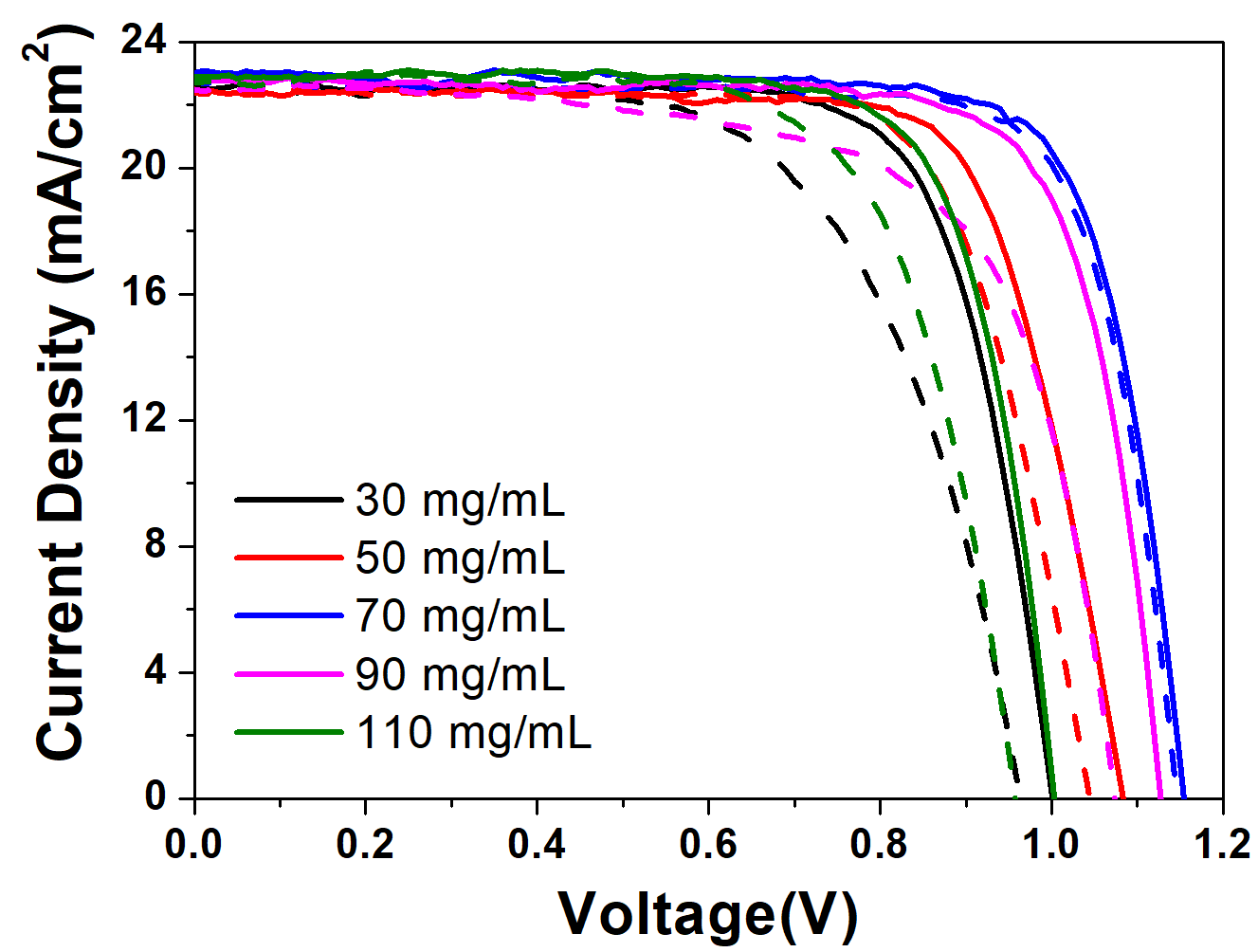
**

**Figure S9.** J-V curves of PSCs using Tetrahydrofuran as a solvent for Spiro-OMeTAD at different concentrations.

**Table S3.** Photovoltaic parameters of PSCs using THF as a solvent for Spiro-OMeTAD at different concentrations.

| **Concentation** | **Scan direction** | **V_OC_ [V]** | **Jsc [mA/cm^2^]** | **FF** | **PCE [%]** |
| --- | --- | --- | --- | --- | --- |
| 30 mg/mL | Rev.  For. | 1.00  0.96 | 22.58  22.84 | 0.748  0.624 | 16.89  13.68 |
| 50 mg/mL | Rev.  For. | 1.08  1.04 | 22.51  22.39 | 0.752  0.742 | 18.28  17.28 |
| 70 mg/mL | Rev.  For. | 1.15  1.14 | 23.03  22.78 | 0.783  0.782 | 20.74  20.31 |
| 90 mg/mL | Rev.  For. | 1.13  1.07 | 22.81  22.49 | 0.773  0.678 | 19.92  16.32 |
| 110 mg/mL | Rev.  For. | 1.01  0.96 | 22.88  22.73 | 0.759  0.705 | 17.54  15.38 |

**Table S4.** The photovoltaic performance parameters of PSCs on FTO substrates

| **Device** | **Scan direction** | **V_OC_ [V]** | **Jsc [mA/cm^2^]** | **FF** | **PCE [%]** |
| --- | --- | --- | --- | --- | --- |
| Spiro-Li in CB | Rev.  For. | 1.11  1.10 | 24.80  24.62 | 0.812  0.801 | 22.35  21.69 |
| Spio-MMPy in CB | Rev.  For. | 1.15  1.12 | 24.64  24.50 | 0.803  0.801 | 22.75  21.98 |
| Spiro-MMPy in THF | Rev.  For. | 1.16  1.12 | 24.68  24.61 | 0.807  0.796 | 23.10  21.94 |


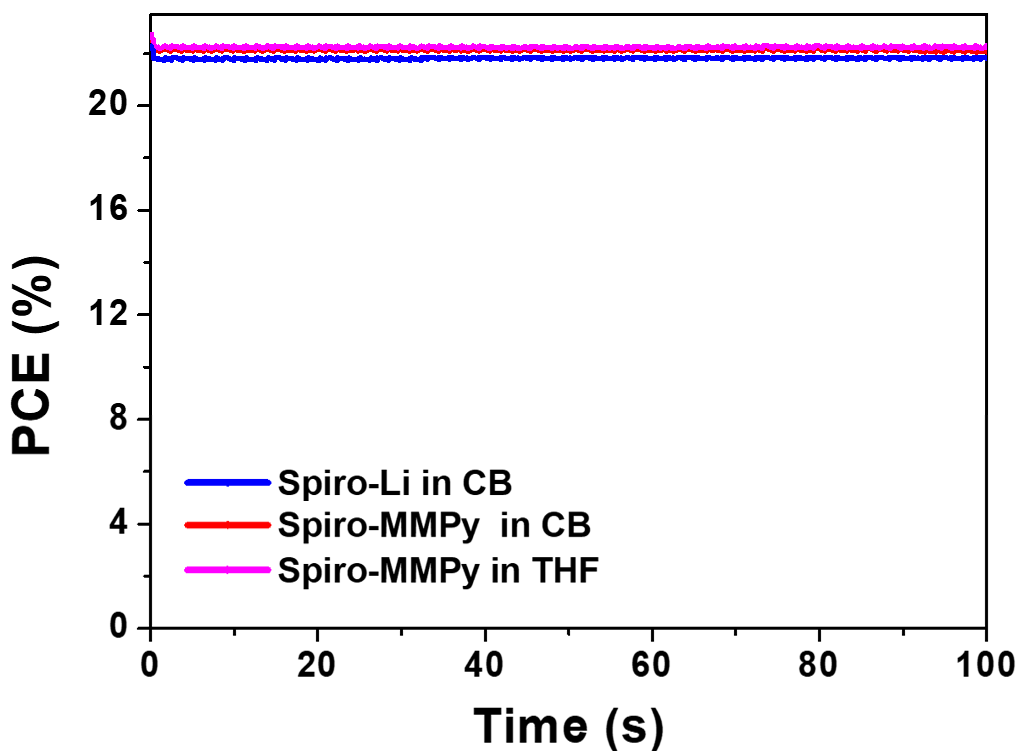


**Figure S10.** Maximum power point tracking (MPPT) results for devices.

**
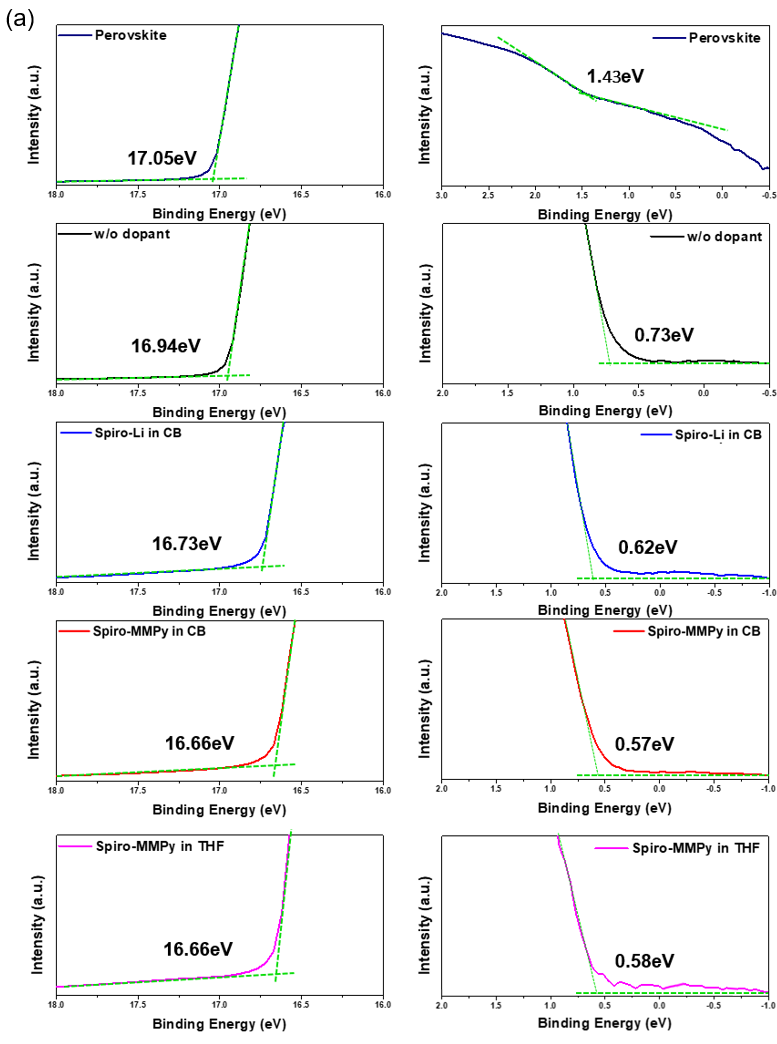
**

**
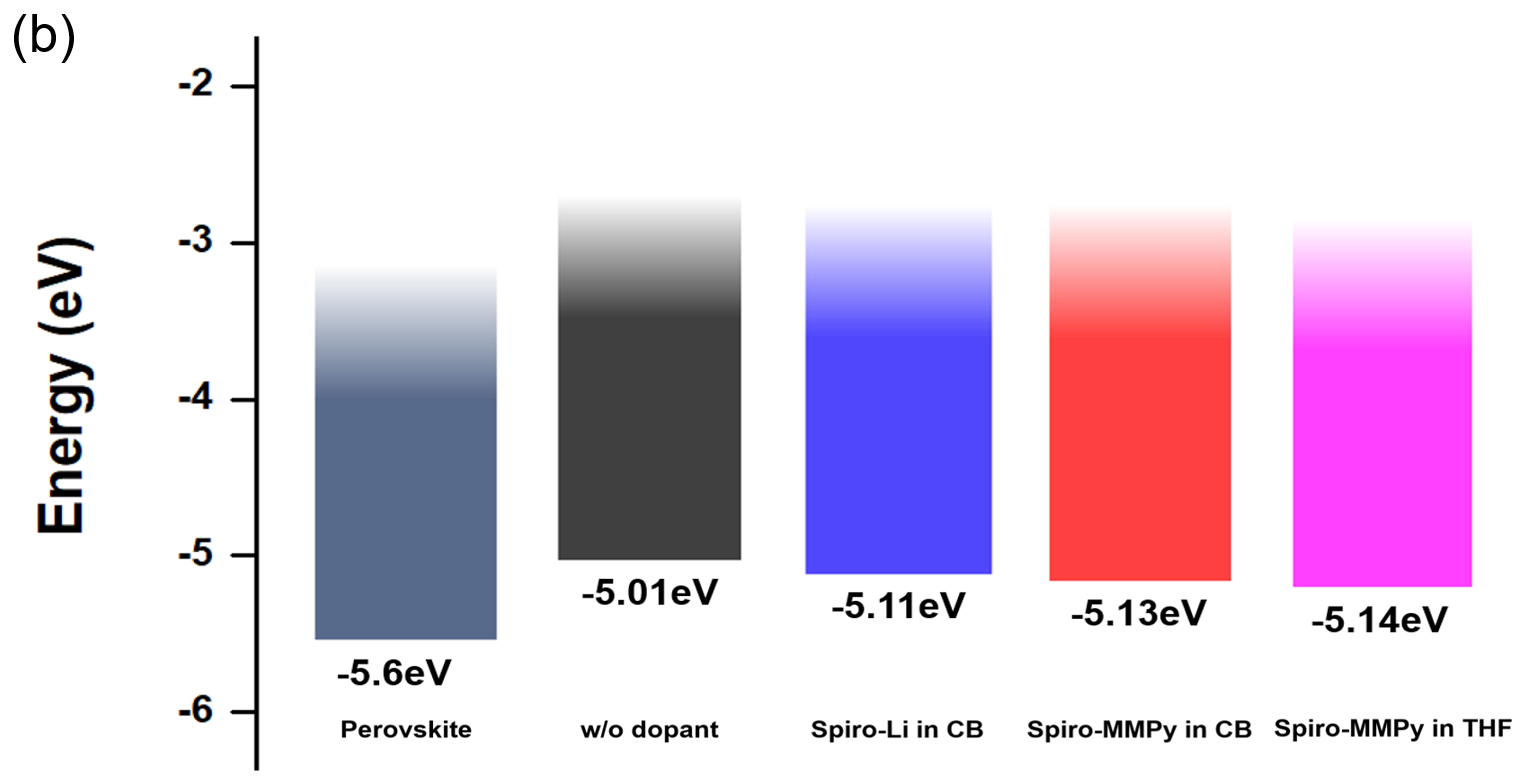
**

**Figure S11.** (a) The cutoff regions of UPS spectra and onset regions of UPS spectra on the logarithmic scale of photoemission intensity of the perovskite film and Spiro-OMeTAD films Their work functions and valence band maximum (VBMs) can be derived. (b) energy level alignments of Perovskite and Spiro-OMeTAD with LiTFSI in CB, MMPyTFSI in CB, MMPyTFSI in THF, and Without Dopants.

**Table S5.** The fitted carrier lifetimes of glass / perovskite / Spiro-OMeTAD samples.

|  | **Tau 1 [ns]** | **Tau 2 [ns]** |
| --- | --- | --- |
| w/o dopant | 20.74±0.06 | 112.12±10.20 |
| Spiro-Li in CB | 3.53±0.45 | 22.29±2.18 |
| Spiro-MMPy in CB | 1.35±0.11 | 14.62±1.58 |
| Spiro-MMPy in THF | 1.26±0.17 | 10.18±1.36 |

**
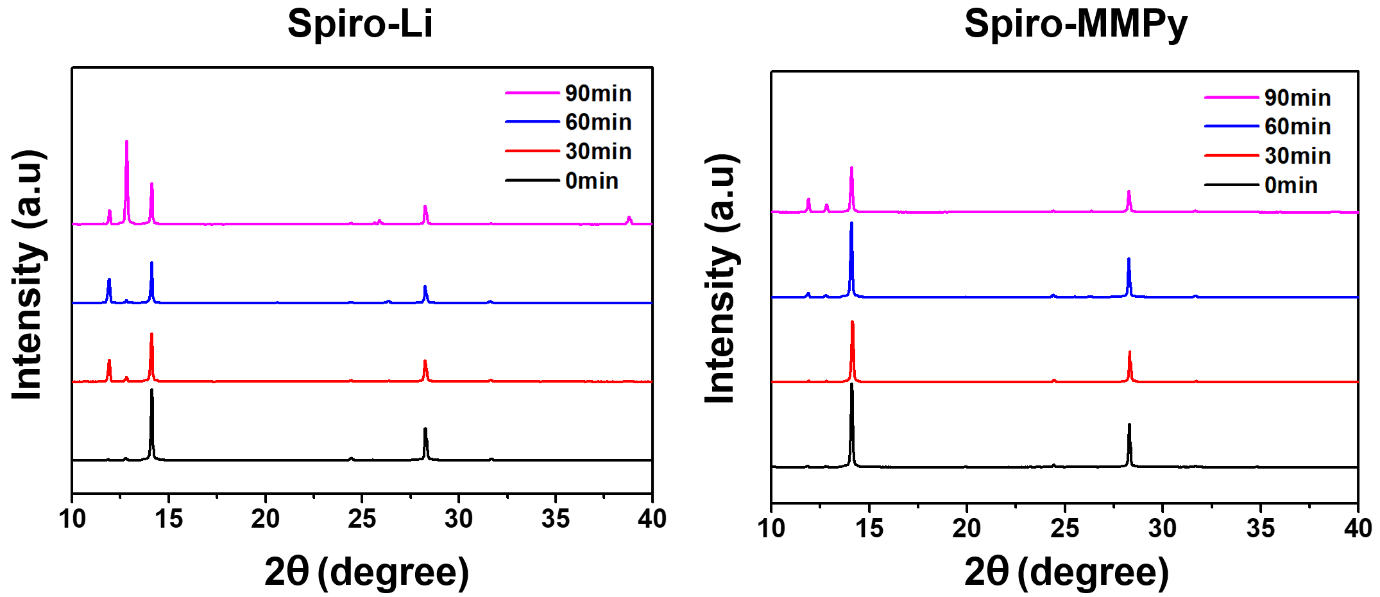
**

**Figure S12.** X-ray Diffraction (XRD) Patterns of Perovskite Films with Spiro-Li and Spiro-MMPy HTLs at 0 min, 30 min, 60 min, and 90 min Under 80% Humidity.

**
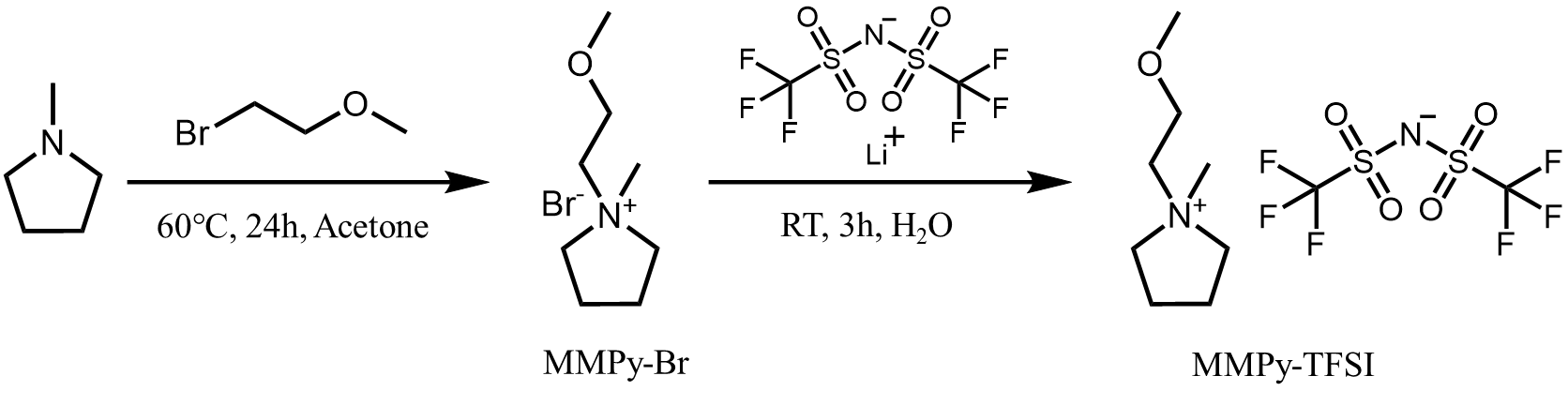
**

**Figure S13**. Synthetic procedure of MMPy-Br and MMPy-TFSI.

**
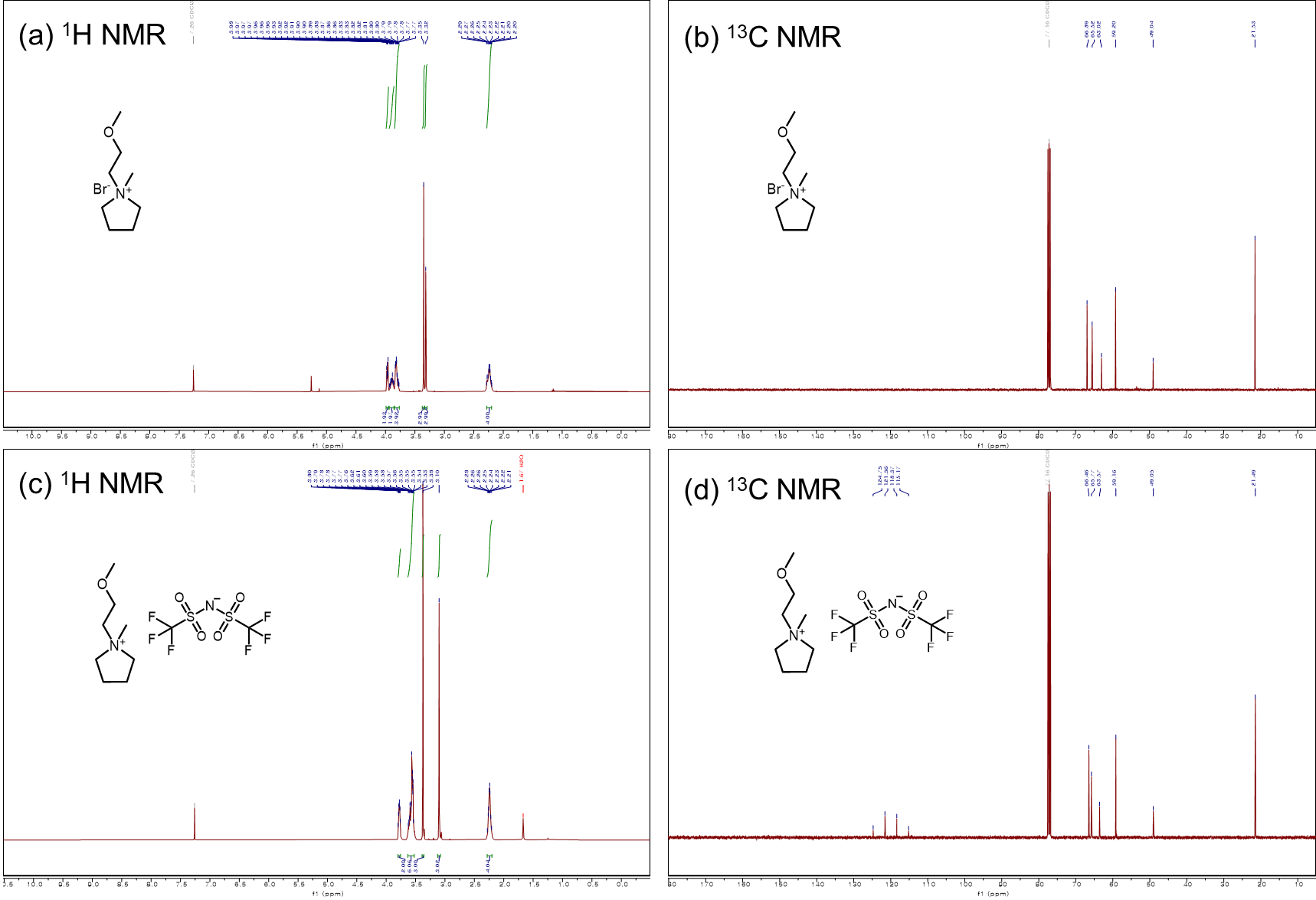
**

**Figure S14.** (a-d) ^1^H NMR and ^13^C NMR spectrum of MMPy-Br and MMPy-TFSI.

**
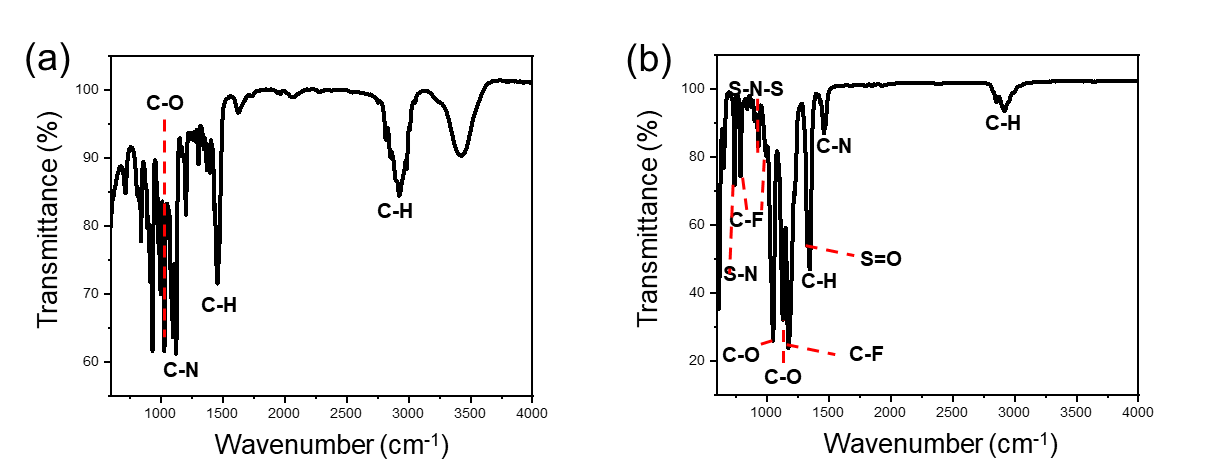
**

**Figure S15.** FTIR spectra of the MMPy-Br and MMPy-TFSI.

(a) :The FTIR spectrum exhibits a band at 2915 cm⁻¹, which is attributed to the asymmetric stretching of CH₃ groups. The band observed at 1464 cm⁻¹ corresponds to the stretching vibration of CH₂ groups. Additionally, bands at 1126 cm⁻¹ and 1030 cm⁻¹ are assigned to the C-O and the C-N stretching vibrations in 1-(2-methoxyethyl)-1-methylpyrrolidine, respectively. (b):The band at 2943 cm⁻¹ is attributed to the asymmetric stretching of CH₃. The first peak at 1464 cm⁻¹ the stretching vibration of CH₂ groups. The band observed at 1349 cm⁻¹ corresponds to the asymmetric SO₂ stretching mode of TFSI, while the subsequent peak at 1329 cm⁻¹ is assigned to the S=O bonding mode of TFSI⁻. The bands at 1132 cm⁻¹ and 1056 cm⁻¹ are assigned to the C-O and the C-N stretching vibrations in 1-(2-methoxyethyl)-1-methylpyrrolidine, respectively. C-F bonding mode of TFSI appears at 1185 cm⁻¹. Sharp peaks at 934 cm⁻¹ and 992 cm⁻¹ are attributed to the asymmetric S-N-S stretching mode and the asymmetric stretching of CF₃, respectively. Two weak peaks at 787 cm⁻¹ and 747 cm⁻¹ correspond to the symmetric bending mode of CF₃ and the S-N stretching mode, respectively.
